# Tumor Immunotherapy–Related Information on Internet-Based Videos Commonly Used by the Chinese Population: Content Quality Analysis

**DOI:** 10.2196/50561

**Published:** 2024-02-07

**Authors:** Chen-xu Ni, Yi-bo Fei, Ran Wu, Wen-xiang Cao, Wenhao Liu, Fang Huang, Fu-ming Shen, Dong-jie Li

**Affiliations:** 1 Shanghai Tenth People’s Hospital Shanghai China

**Keywords:** immunotherapy, internet videos, quality, misinformation, health informatics, Chinese

## Abstract

**Background:**

Tumor immunotherapy is an innovative treatment today, but there are limited data on the quality of immunotherapy information on social networks. Dissemination of misinformation through the internet is a major social issue.

**Objective:**

Our objective was to characterize the quality of information and presence of misinformation about tumor immunotherapy on internet-based videos commonly used by the Chinese population.

**Methods:**

Using the keyword “tumor immunotherapy” in Chinese, we searched TikTok, Tencent, iQIYI, and BiliBili on March 5, 2022. We reviewed the 118 screened videos using the Patient Education Materials Assessment Tool—a validated instrument to collect consumer health information. DISCERN quality criteria and the JAMA (*Journal of the American Medical Association*) Benchmark Criteria were used for assessing the quality and reliability of the health information. The videos’ content was also evaluated.

**Results:**

The 118 videos about tumor immunotherapy were mostly uploaded by channels dedicated to lectures, health-related animations, and interviews; their median length was 5 minutes, and 79% of them were published in and after 2018. The median understandability and actionability of the videos were 71% and 71%, respectively. However, the quality of information was moderate to poor on the validated DISCERN and JAMA assessments. Only 12 videos contained misinformation (score of >1 out of 5). Videos with a doctor (lectures and interviews) not only were significantly less likely to contain misinformation but also had better quality and a greater forwarding number. Moreover, the results showed that more than half of the videos contain little or no content on the risk factors and management of tumor immunotherapy. Overall, over half of the videos had some or more information on the definition, symptoms, evaluation, and outcomes of tumor immunotherapy.

**Conclusions:**

Although the quality of immunotherapy information on internet-based videos commonly used by Chinese people is moderate, these videos have less misinformation and better content. Caution must be exercised when using these videos as a source of tumor immunotherapy–related information.

## Introduction

### Background

Tumor immunotherapy is an innovative treatment today. After the implementation of China's new medical insurance rates in 2022, the monthly treatment cost of immunotherapy has entered the “thousand era,” which greatly improves the accessibility of drugs. However, tumor immunotherapy has obvious uncertainty and complexity [1]. Accurate transmission of immunotherapy information to the population is important to the survival and quality of life of patients with cancer [2]. The study found that patients were open to video education and found it helpful and worth watching [3].

The world’s population is increasingly referring to health-related internet-based information as it represents an easily accessible educational tool [4,5]. The Chinese population, overseas Chinese individuals, and people who master Chinese worldwide prefer web-based video applets or websites, such as videos on TikTok, Tencent, iQIYI, or BiliBili [6]. These sites, similar to YouTube, are popular for their rich content, convenient log-in methods, quick sharing, and 24-hour multiplatform seamless application experiences. Recently, the originality, interactivity, and sociable nature of TikTok and BiliBili have provided the younger generation a better user experience and sense of engagement while seeking health information [7]. The penetration and usage of TikTok and BiliBili are also on the rise among some older age groups [8]. However, the medical content available on the internet is controversial and has not been properly examined. Di Bello et al [9] reported that YouTube videos have contributed to the spread of misinformation by underestimating the role of information on immunotherapy for urological tumors in a multimodality approach and missing the findings of published clinical trials. Not only were audiences not availing of accurate therapy, but also they were opting for therapies that may be harmful, which could lead to other complications [10,11].

### Objectives

This study aims to report an evaluation of the quality, reliability, and content of videos related to tumor immunotherapy on the internet among the Chinese population. Our findings could serve as a guide for health care providers and awareness campaigns.

## Methods

### Ethical Considerations

Ethics approval was not required as this descriptive study was conducted by examining publicly accessible videos on the internet. Also, no human participants or animals were included in this study. The study data are anonymous. This study was registered in the Chinese Clinical Trial Registry (ChiCTR2400081071).

### Search Strategy and Data Collection

Using the keyword “肿瘤免疫治疗” (“tumor immunotherapy” in Chinese), we searched TikTok, Tencent, iQIYI, and BiliBili on March 5, 2022, which yielded 1820, 395, 400, and 1000 results for each search, respectively. The videos were sorted in accordance with the video's default “the most viewed” sorting parameter, and the first 50 videos per website were evaluated.

### Inclusion and Exclusion Criteria

**Figure 1:**
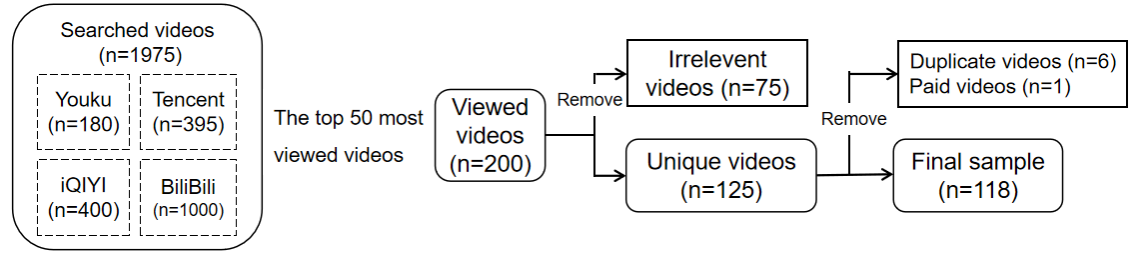
Search strategy and video screening procedure.

A total of 200 videos were considered from all the searches. Duplicate videos, paid videos, and videos not related to tumor immunotherapy were excluded. After the screening, we obtained 118 videos for further data extraction and analysis (Figure 1).

### Variables Extracted

Basic information obtained included the URL, video duration, likes, forwarding number, subscription, comments, and upload date. Profiles of the uploaders were recorded and classified under 5 categories: lectures, interviews, health-related animations, academic institutions or universities, and news agencies. The extracted data were recorded in Excel (Microsoft Corp).

### Scoring System

The videos were evaluated independently by 2 authors (CN and Y-BF). The raters were blinded to each other's ratings (they could not consult each other). We reviewed the screened 118 of 3615 videos on TikTok, Tencent, iQIYI, and BiliBili on “tumor immunotherapy,” using the Patient Education Materials Assessment Tool (PEMAT)—a validated instrument for obtaining consumer health information (Multimedia Appendix 1) [12]. Moreover, we adopted 6 questions from Goobie et al [13] to evaluate the videos’ content. These 6 questions ask to what degree a video addresses the definition of a disease, its signs and symptoms, risk factors, evaluation, management, and outcomes. Each aspect was scored on a 3-item scale: 0=not addressed, 1=partially addressed, and 2=sufficiently addressed.

The DISCERN quality criteria [14] and the JAMA (*Journal of the American Medical Association*) Benchmark Criteria [15] were used for assessing the quality and reliability of the health information. The modified version of the original DISCERN questionnaire was used to assess the reliability and quality of the health information. It consists of five questions, each with a “yes/no” answer (yes=1 point; no=0 points; maximum score=5): (1) Is the video clear and complete? (2) Are reliable sources of information used? (3) Is the information presented balanced and unbiased? (4) Are additional sources of information listed for reference? (5) Are uncertain areas mentioned? The JAMA assessment is used to evaluate web-based videos and resources on the basis of 4 criteria: authorship, attribution, disclosure, and currency (1 point each).

- Authorship (1 point): the video should include authors, contributors, and contact information.
- Attribution (1 point): the references and sources should be listed properly.
- Disclosure (1 point): conflicts of interest, financing, sponsorship, advertising, support, and video ownership should be disclosed.
- Currency (1 point): the dates on which the videos were published and updated should be indicated.

After the scores are calculated, a score of 4 indicates that the source is of high quality.

We assessed the presence of misinformation using an analogous 5-point Likert scale [16,17]. Videos were independently coded by 2 authors with random coding checks to verify intercoder reliability. Each video was rated separately, and its mean score was calculated.

### Statistical Analysis

The mean, median, IQR, and SD were used as descriptive statistics for continuous variables. To identify differences among the variables extracted, the Mann-Whitney *U* test was performed. The intraclass correlation coefficient was determined to ensure interrater reliability. A *P* value of less than .05 was considered significant. Statistical analysis was performed by using the GraphPad Prism 8 (GraphPad Software, Inc).

## Results

The 118 videos about tumor immunotherapy mostly uploaded by channels dedicated to lectures, health-related animations, and interviews (Table 1; median length 5 minutes; 93, 79% uploaded in and after 2018). The median forwarding number and number of likes was 12 and 15, respectively. However, the median understandability and actionability of the videos were 71% and 71%, respectively. Overall, the quality of information was moderate to poor in 54% of videos (overall DISCERN scores of 1-3 out of 5) and 64% of videos (overall JAMA scores of 1-2 out of 4).

Only 12 videos contained misinformation (score >1 out of 5). Videos with a doctor (published by channels dedicated to lectures and interviews) not only were significantly less likely to contain misinformation but also had better quality and a greater forwarding number. Videos on Tencent and BiliBili had lesser misinformation than TikTok and iQIYI. Regarding DISCERN criteria and JAMA Benchmark Criteria, the quality of information on TikTok and iQIYI was higher than that on BiliBili and Tencent.

**Figure 2:**
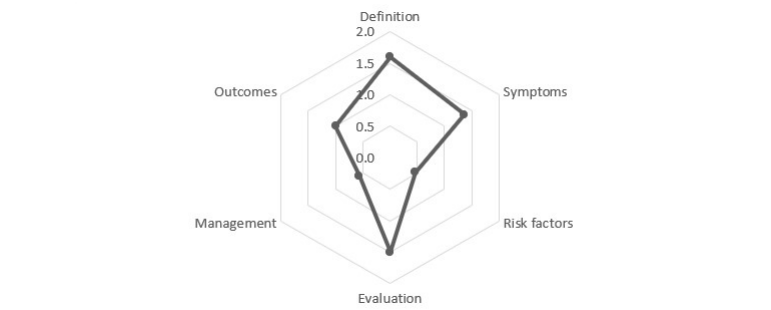
Completeness of content in internet-based videos.

Moreover, our results show that more than half of the videos contain little or no content on the risk factors and management of tumor immunotherapy. Overall, over half of the videos had some or more information on the definition, symptoms, evaluation, and outcomes of tumor immunotherapy (Table 2). The overall scores for all internet videos are presented in Figure 2.

**Table 1:** Characteristics of internet-based videos about immunotherapy.

Characteristics		Value
Length of the video (minutes), median ( IQR)		5.0 (1.0-118.2)
**Year of publication of the video, n (%)**		
	Before 2018	25 (21)
	2018 and after	93 (79)
Forwarding number, median (range)		12 (0-364)
Likes, median (range)		15 (0-1613)
Comments, median (range)		0 (0-215)
Subscription, median (range)		0 (0-1473)
**Publisher type, n (%)**		
	Lecture	51 (43)
	Interview	22 (19)
	News agency	9 (8)
	Health-related animation	33 (27)
	Academic institution or university	3 (3)
**Overall DISCERN scores, n (%)**		
	1	2 (2)
	2	8 (7)
	3	44 (37)
	4	33 (28)
	5	6 (5)
**DISCERN scores, mean (SD)**		
	TikTok	3.0 (1.0)
	Tencent	2.5 (1.8)
	iQIYI	3.3 (0.6)
	BiliBili	2.7 (1.6)
**PEMAT^a^ scores (%), median (IQR)**		
	Understandability	75 (22-100)
	Actionability	71 (0-100)
**Misinformation score, n (%)**		
	1	6 (5)
	2	5 (4)
	3	0 (0)
	4	1 (1)
	5	0 (0)
**Misinformation score, mean (SD)**		
	Lecture	0.1 (0.6)
	Interview	0.2 (0.5)
	Health-related animation	0.3 (0.7)
**Misinformation score, mean (SD)**		
	TikTok	0.4 (0.8)
	Tencent	0.09 (0.3)
	iQIYI	0.2 (0.8)
	BiliBili	0.1 (0.4)
**JAMA^b^ overall score, n (%)**		
	1	17 (14)
	2	47 (40)
	3	16 (13)
	4	4 (3)
**JAMA score, mean (SD)**		
	TikTok	1.8 (0.8)
	Tencent	1.3 (1.4)
	iQIYI	1.9 (0.7)
	BiliBili	1.3 (1.1)

^a^PEMAT: Patient Education Materials Assessment Tool.

^b^JAMA: *Journal of the American Medical Association*.

**Table 2:** Completeness of the content of videos on the internet.

Content	Definition, n (%)	Symptoms, n (%)	Risk factors, n (%)	Evaluation, n (%)	Management, n (%)	Outcomes, n (%)
No content (0 points)	17 (14)	15 (13)	53 (45)	7 (6)	46 (39)	30 (25)
Little content (0.5 points)	8 (7)	15 (13)	14 (12)	7 (6)	12 (10)	8 (7)
Some content (1 point)	12 (10)	19 (16)	20 (17)	40 (34)	35 (30)	50 (43)
Most content (1.5 points)	25 (21)	25 (21)	17 (14)	26 (22)	11 (9)	17 (14)
Extensive content (2 points)	60 (48)	44 (37)	14 (12)	38 (32)	14 (12)	13 (11)

## Discussion

We screened 118 videos on “tumor immunotherapy” from TikTok, Tencent, iQIYI, and BiliBili commonly used by the Chinese population. Chinese websites or applets uploaded videos related to tumor immunotherapy for the first time in 2011, and the number of videos has significantly increased since 2018. The median duration of the videos was 5 minutes, which is acceptable to the public.

Numerous studies have evaluated videos on YouTube only and not on other networks [18-20]. Our study evaluated information about tumor immunotherapy on the most popular Chinese websites or applets, using validated instruments to evaluate the quality of information. Videos on BiliBili and TikTok had a significantly greater forwarding number and likes than those on iQIYI and Tencent; a possible reason is that there is no advertisement played before videos on BiliBili and TikTok.

Health care providers should recommend trustworthy sources of information to patients and should actively participate in social media for dissemination of evidence-based medicine. There is a great need for accurate tumor immunotherapy–related content that is also understandable and actionable. Suggestions for content creators include discussing both the benefits and risks of management alternatives, refraining from the use of medical terminology, and presenting the viewer with clear action items. Meanwhile, patients should be wary of internet-based videos. Misinformation, albeit well-intentioned, may be disseminated when a poorly informed patient advises others. Patients should talk to their physicians not only about immunotherapy but also their need for more information.

In conclusion, although the quality of tumor immunotherapy–related information on internet-based videos commonly used by Chinese people is moderate, it has less misinformation and better content. Caution must be exercised when using these videos as a source of tumor immunotherapy–related information.

## Appendix

Multimedia Appendix 1 Full scores for PEMAT measures of understandability and actionability. PEMAT: Patient Education Materials Assessment Tool.

## Abbreviations

JAMA: *Journal of the American Medical Association*
PEMAT: Patient Education Materials Assessment Tool

## Data Availability

The data sets generated during or analyzed during this study are available from the corresponding author on reasonable request.
