# Correction: Tumor Immunotherapy–Related Information on Internet-Based Videos Commonly Used by the Chinese Population: Content Quality Analysis

**DOI:** 10.2196/59671

**Published:** 2024-05-02

**Authors:** Chen-xu Ni, Yi-bo Fei, Ran Wu, Wen-xiang Cao, Wenhao Liu, Fang Huang, Fu-ming Shen, Dong-jie Li

**Affiliations:** 1 Shanghai Tenth People’s Hospital Shanghai China

In “Tumor Immunotherapy–Related Information on Internet-Based Videos Commonly Used by the Chinese Population: Content Quality Analysis” (JMIR Form Res 2024;8:e50561) the authors made one addition.

The following sentence was added to the “Ethical Considerations” section under “Methods”:

This study was registered in the Chinese Clinical Trial Registry (ChiCTR2400081071).

The correction will appear in the online version of the paper on the JMIR Publications website on May 2, 2024 together with the publication of this correction notice. Because this was made after submission to PubMed, PubMed Central, and other full-text repositories, the corrected article has also been resubmitted to those repositories.

