# Supplementary material for: Tumor Immunotherapy–Related Information on Internet-Based Videos Commonly Used by the Chinese Population: Content Quality Analysis
Source: JMIR Form Res. 2024 Feb 7;8:e50561. doi: 10.2196/50561 (PMC10882464; doi:10.2196/50561)
Supplement: Multimedia Appendix 1 [file formative_v8i1e50561_app1.docx]

**Supplementary Table 1.** Full scores for PEMAT measures of understandability (consumers of diverse backgrounds and varying levels of health literacy can process and explain key messages) and actionability (consumers of diverse backgrounds and varying levels of health literacy can identify what they can do based on the information presented).

| **Item** | **PEMAT measure** | **Responses (%)** | | |
| --- | --- | --- | --- | --- |
|  |  | **Disagree** | **Agree** | **NA** |
| ***Understandability items by topic*** | | | | |
| Content | | | | |
| 1 | Purpose evident | 37 | 63 | – |
| Word choice and style | | | | |
| 2 | Material uses everyday language | 37 | 63 | – |
| 3 | Medical terms defined | 34 | 66 | – |
| 4 | Material uses active voice | 26 | 74 | – |
| Organization | | | | |
| 5 | Material broken down into short sections | 27 | 41 | 32 |
| 6 | Sections have informative headers | 45 | 29 | 26 |
| 7 | Presented in logical sequence | 23 | 77 | – |
| 8 | Provides summary | 54 | 21 | 25 |
| Layout and design | | | | |
| 9 | Visual cues to draw attention to key points | 18 | 21 | 61 |
| 10 | Text easy to read | 6 | 39 | 55 |
| 11 | Hear words clearly | 11 | 83 | 6 |
| Use of visual aids | | | | |
| 12 | Illustrations/photos are clear | 7 | 31 | 62 |
| 13 | Simple/clear tables | 10 | 1 | 89 |
| ***Actionability items by topic*** | | | | |
| 14 | Identifies at least one action | 53 | 47 | – |
| 15 | Addresses user directly | 7 | 9 | 84 |
| 16 | Breaks down action into steps | 76 | 24 | – |
| 17 | Explains how to use | 63 | 37 | – |

PEMAT = Patient Education Materials Assessment Tool; NA = not applicable.
